# Audiomotor Temporal Recalibration Modulates Decision Criterion of Self-Agency but Not Perceptual Sensitivity

**DOI:** 10.3389/fpsyg.2021.580441

**Published:** 2021-04-26

**Authors:** Yoshimori Sugano

**Affiliations:** Department of Business and Marketing, Faculty of Commerce, Kyushu Sangyo University, Fukuoka, Japan

**Keywords:** temporal recalibration, delayed auditory feedback, sense of agency, sensorimotor coordination, signal detection theory

## Abstract

Exposure to delayed sensory feedback changes perceived simultaneity between action and feedback [temporal recalibration (TR)] and even modulates the sense of agency (SoA) over the feedback. To date, however, it is not clear whether the modulation of SoA by TR is caused by a change in perceptual sensitivity or decision criterion of self-agency. This experimental research aimed to tease apart these two by applying the signal detection theory (SDT) to the agency judgment over auditory feedback after voluntary action. Participants heard a short sequence of tone pips with equal inter-onset intervals, and they reproduced it by pressing a computer mouse. The delay of each tone pip after the mouse press was manipulated as 80 (baseline) or 180 ms (delayed). Subsequently, the participants reproduced it, in which the delay was fixed at 80 ms and there was a 50% chance that the computer took over the control of the tone pips from the participants. The participants’ task was to discriminate who controlled the tone pips and to judge synchrony between tone pips and mouse presses. Results showed that the modulation of the SoA by the TR is caused by a shift in the decision criterion but not in the perceptual sensitivity of agency.

## Introduction

Delay in the sensory feedback following our voluntary action disrupts our smooth interaction with the environment (e.g., Wegner and Wheatley, 1999; Cunningham et al., 2001b; Engbert et al., 2008; Morice et al., 2008). One example is a telecommunication delay over the cellphone, which disrupts one’s smooth conversation with his or her partner. Another example is a response delay from a personal computer, which disrupts one’s rapid and smooth operations on the computer. However, if we are exposed to the feedback delay for a while, we get used to it and even become unaware of it. As a result, we can return to a state of smooth sensorimotor coordination over the device with delayed feedback—we have adapted to the delay. When we are adapting to the delay, what is going on in our sensorimotor system?

Researchers have focused on two different types of change in our sensorimotor system with the adaptation to the delay: a change in the sense of control over the outcome of an action and a change in the perceived timing of the action and the outcome. The sense that I am causing an action is called the sense of agency (SoA) (Ghallagher, 2000; Wolpe and Rowe, 2014;

Imaizumi and Asai, 2015), which sometimes includes a sense of control over the consequences of an action (Haggard, 2005; Chambon et al., 2013; Kawabe, 2015; Moore, 2016). The perceived timing of action and outcome is one of the key factors of the SoA. The SoA is disrupted if there is a delay between a voluntary action and a sensory feedback (Shanks and Dickinson, 1991; Franck et al., 2001; Sato and Yasuda, 2005; Farrer et al., 2013; Kawabe et al., 2013; Rohde et al., 2014b; Timm et al., 2014; Wen et al., 2015; for review, Wen, 2019), which clearly indicates that the perceived temporal proximity between voluntary actions and their feedback is a strong cue for agency.

**FIGURE 1:**
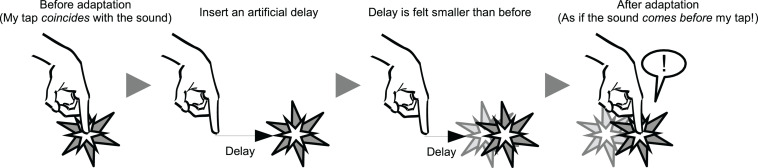
Schematic illustration of the temporal recalibration (TR) and degraded sense of agency (SoA) after the TR. After exposure to an artificial delay between a voluntary action (i.e., a tap) and a subsequent external sensory feedback (i.e., a sound), a perception of subjective simultaneity between the action and the feedback is modulated drastically in a way that an objectively synchronous sensory feedback is perceived as if it comes before the action.

On the other hand, it has been shown that the perceived simultaneity between actions and their consequences can be recalibrated after exposure to delayed sensory feedback (Stetson et al., 2006; Heron et al., 2009; Sugano et al., 2010). This is known as temporal recalibration (TR) (Vroomen et al., 2004; Navarra et al., 2005; Harrar and Harris, 2008) or lag adaptation (Fujisaki et al., 2004; Miyazaki et al., 2006). With these two phenomena together, we can expect that TR modulates the SoA over voluntary action, as the perceived simultaneity between voluntary action and its feedback is a primary cue for agency. In fact, this is the case. Anecdotal evidence indicates that the consequence of one’s voluntary action is perceived “before” the action takes place, even if it is delivered “after” the action, after exposure to delayed sensory feedback (Cunningham et al., 2001a; Stetson et al., 2006), suggesting that the SoA is modulated after the TR (see Figure 1 for graphical explanation).

Recent experimental research also supports this claim. It has been demonstrated that the SoA over non-delayed sensory feedback decreases after TR (Timm et al., 2014; Haering and Kiesel, 2015, 2016; Imaizumi and Asai, 2015; Imaizumi and Tanno, 2019). This happens because the SoA depends on subjective (not veridical) simultaneity between action and feedback, which the TR does modulate.

However, currently, it is still not clear whether the modulation of the SoA by TR is due to a change in perceptual sensitivity or a change in decision criterion of agency. This point is important in shedding light on the mechanism of SoA modulation by TR. If it were caused by a change in perceptual sensitivity, it would be a product of an *early* stage of processing, which is perceptual (e.g., Webster, 2011). On the other hand, if it were caused by a change in the decision criterion, it would be a product of a *late* stage of processing, which is either perceptual (e.g., a change in the balance of pooling neurons from low-level ones; Cai et al., 2012) and/or cognitive (e.g., a change in the criterion for categorizing events; Yarrow et al., 2011, 2013).

The present study tackled this issue. To tease apart these two, I utilized an experimental paradigm using a finger-tapping task, which was first introduced by Knoblich and Repp (Repp and Knoblich, 2007; Knoblich and Repp, 2009). Analyzing the agency judgment (AJ) data using the signal detection theory (SDT; Green and Swets, 1966), the sensitivity and response bias in the judgment of the SoA can be separated (Asai, 2017).

The perceptual and/or cognitive nature of TR has been investigated by previous psychophysical and neuropsychological studies. Fujisaki et al. (2004) found that the audiovisual TR occurs using a stream-bounce illusion that is thought to be free from a cognitive bias. Focusing on the generalization and decay of the effect, Kennedy et al. (2009) tested whether visuomotor TR is a product of instrumental learning (which decays fast) or perceptual learning (which decays slowly). They found that visuomotor TR shows slow decay, suggesting that it was perceptual learning rather than instrumental learning (i.e., operant conditioning). Moreover, as the sensorimotor TR transfers to the other sensorimotor pairings (Heron et al., 2009; Sugano et al., 2010), it could not be a simple criterion change of simultaneity, which would be restricted to the adapted sensorimotor parings (di Luca et al., 2009; Vroomen and Keetels, 2010). These findings suggest that TR has a perceptual and not a cognitive origin.

On the other hand, Simon et al. (2017) investigated audiovisual TR using event-related potentials (ERPs) and found that relatively late ERP components (>125 ms) were affected by the temporal order of the audiovisual pairs (i.e., audio-leading vs. visual-leading) in the preceding trial, suggesting that it reflects a late sensory and/or decisional processing rather than early sensory processing. Notably, they investigated rapid TR, which occurs after only one trial (Wozny and Shams, 2011; van der Burg et al., 2013, 2014, 2015; de Niear et al., 2017). Therefore, the ERP evidence by Simon et al. (2017) holds true for rapid TR, but it is not clear whether it is related to cumulative TR.

As these findings point toward the perceptual nature of TR, it is natural to predict that the modulation of the SoA by TR also functions at the perceptual level. If this were true, TR would modulate the perceptual sensitivity of agency. However, there have been alternative arguments that favor the cognitive origin of cross-sensory (i.e., audiovisual) TR (Yarrow et al., 2011; van der Burg et al., 2015), which suggest that TR may be a product of the change in decision criterion about simultaneity. In addition, as the SoA is thought to be a product of complex reasoning about self-agency (e.g., inference about cause–effect relationship; Synofzik et al., 2008), we can also foresee that the modulation of the SoA by TR works at the decisional level. If this were true, TR would modulate the response bias in AJ. Currently, however, there is no decisive evidence regarding which is true.

Before exploring the modulation of the SoA by TR, a theoretical framework connecting the simultaneity perception with the agency perception should be introduced. The point of perceived simultaneity is thought to be extended in time in order to integrate various sensory inputs into a coherent percept (e.g., psychological present; Michon, 1978), which is often termed as a temporal window of simultaneity (TWS; Varela, 1999; Slutsky and Recanzone, 2001; Fujisaki and Nishida, 2005; van Wassenhove et al., 2007; Tanaka et al., 2011; Chen and Vroomen, 2013; Rohde et al., 2014a; Wallace and Stevenson, 2014; Noel et al., 2016; Simon et al., 2017). Likewise, the point of perceived agency is thought to be extended in time, which can be referred to as a temporal window of agency (TWA; Farrer et al., 2013; Rohde et al., 2014b; Timm et al., 2014). As the perceived simultaneity between voluntary action and sensory feedback is a strong sensorimotor cue for the SoA (Franck et al., 2001; Sato and Yasuda, 2005; Rohde et al., 2014b; Timm et al., 2014), it is highly likely that the modulation of the TWA co-occurs with the modulation of the TWS. Yet, it is still not clear how they are correlated. As has been shown that TR shifts the midpoint of the TWS between action and feedback (Stetson et al., 2006; Heron et al., 2009; Sugano et al., 2010) and even modulates the width of the TWS (Winter et al., 2008; Keetels and Vroomen, 2012; Sugano et al., 2016), it is expected that TR also modulates the midpoint and/or the width of the TWA.

**FIGURE 2:**
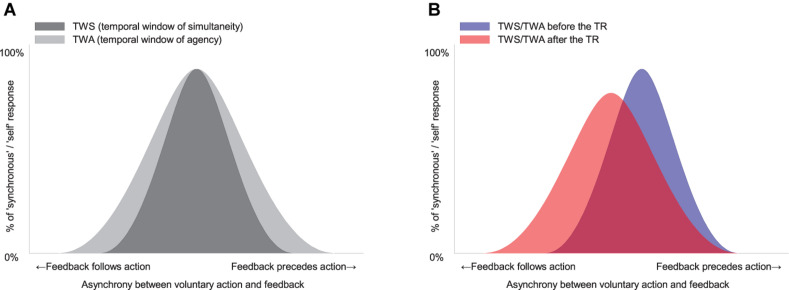
The model and the predictions about a change in sense of agency (SoA) after temporal recalibration (TR). **(A)** Hypothetical temporal window of simultaneity (TWS) and of agency (TWA) between voluntary action and sensory feedback. Note that the width of TWA is wider than the width of TWS. **(B)** The TR might modulate a midpoint and/or a width of the TWS and TWA.

Figure 2A illustrates a simple model showing how the TWS relates to simultaneity judgment (SJ) and how the TWA relates to AJ. In this figure, both windows have been drawn as symmetrical Gaussian curves for simplicity. In reality, however, the tolerance zone of subjective simultaneity in the TWS might be asymmetrical due to a cause–effect relationship or a different processing speed in each sensory modality (Franck et al., 2001; Sato and Yasuda, 2005; van Wassenhove et al., 2007; Rohde and Ernst, 2012; Yarrow et al., 2013; Rohde et al., 2014a, b; Timm et al., 2014; for review, Rohde and Ernst, 2016). Additionally, it is of note that the width of the TWA is wider than the width of the TWS in the figure. This is because it has been shown that the width of the TWA is larger at the movement-lead side than that of the TWS (Rohde et al., 2014b).

Figure 2B illustrates how TR modulates the midpoint and/or the width of the TWS and/or the TWA. Critically, the parameters characterizing the shape of the window (i.e., midpoint and width) may relate to the parameters derived from the SDT analysis (response bias and sensitivity, respectively). The midpoint of the window may influence the decision criterion (response bias) in the SJ and the AJ. The width of the window may influence the perceptual sensitivity in the SJ and the AJ.

## Materials and Methods

### Participants

Twenty-two students from Kyushu Sangyo University participated in the experiment from April to October 2018. The sample size was determined by conventions of standard psychophysical experiments (i.e., ∼20; Klatzky et al., 2017). All participants had normal hearing and normal or corrected-to-normal vision. They were all right-handed by self-report. Written informed consent was obtained from each participant. The experiment was approved by the Local Ethics Committee of Kyushu Sangyo University and followed the Declaration of Helsinki.

After analyzing the data preliminarily, four participants (all males, mean age = 22.8 years, range = 19–30 years) were excluded from the main analysis, as their data showed several irregularities (15–30%) according to the predetermined criteria regarding tapping stability (see the “Results” section) compared with the other participants (<10%). The data from the remaining 18 participants (four females, mean age = 20.2 years, range = 19–22 years) were analyzed.

### Stimuli and Apparatus

Experiments were conducted in a small dimly lit and quiet booth, where the background noise level was approximately 35 dB(A). Participants sat at a desk, with a 17-inch CRT kept at approximately 60 cm viewing distance, running with a 100-Hz refresh rate. The CRT was connected to a general personal computer (Dell Precision T3400) that was controlled by E-prime 2.0 software (Psychology Software Tools, Inc.). A pair of headphones (Sony MDR-CD900ST), a special gaming mouse with a high temporal resolution (Logitech G300, with 2-ms polling interval), and a dedicated response box (Psychology Software Tools, Inc.) were also connected to the computer. The auditory stimulus was a 2,000-Hz pure tone pip (30-ms duration with 2-ms rise/fall ramps). When a participant pressed the mouse, the tone was presented *via* headphones at 78 dB(A). White noise was continuously presented *via* a speaker in front of the participant at 45 dB(A) to mask the faint sound of mouse presses. The timing of the audio output and the mouse press detection was verified by a multiple-trace oscilloscope (PicoScope 2203, Pico Technology Ltd.).

### Design and Procedure

A four within-subjects factorial design was used: the feedback delay (80 ms as “baseline” vs. 180 ms as “delayed”), the controlling agent (self-controlled vs. computer-controlled), the interstimulus interval (ISI) of tone sequence (500, 600, and 700 ms), and the number of tones (4, 5, 6, and 7). These four factors yielded 48 unique conditions. Each condition was presented twice, resulting in 96 trials in total.

The experiment consisted of two blocks of two sessions. Each session consisted of 24 trials. The feedback delay was organized into blocks and was constant within each block. The execution order of the blocks was counterbalanced across participants. To minimize a carryover effect between the conditions, a rest period of at least 10 min was inserted between the blocks. The other three factors varied randomly within each session. A short rest (∼1 min) was also inserted between the sessions within a block. The experiment lasted approximately 1.5 h including instruction, practice sessions, main sessions, and debriefing.

One trial consisted of three phases: listening to a model sequence of tones (listening phase), reproducing it by pressing a computer mouse with exposure to delayed feedback (adaptation phase), and reproducing it and judging the agency and simultaneity (test phase). During the listening phase, the participants heard the model sequence of tones with a constant ISI. The ISI and the number of tones were varied randomly across trials. Immediately after the listening phase, the adaptation phase began.

During the adaptation phase, participants reproduced the model sequence of tones by pressing the computer mouse. The delay in tone onset after the participant’s mouse press was manipulated as either 80 (“baseline” condition) or 180 ms (“delayed” condition). Immediately after the adaptation phase, the test phase began.

During the test phase, the participants did the reproduction task again. However, this time, the tone was controlled by either the participants (self-controlled trial) or the computer (computer-controlled trial). As the self-controlled and computer-controlled trials were presented in a random order and the number of trials for each condition was equal, the probability of each condition being presented was 50%. In both trials, the delay in tone onset after the participant’s mouse press was fixed at 80 ms at the initial two tones. However, the computer took over the control of the tone output from the third to the last mouse press in computer-controlled trials. In self-controlled trials, the participants continued to control the output of tones with the fixed 80-ms delay.

Immediately after that, the participants made two-alternative forced choice (2AFC) judgments about the agency (AJ) over the tone output and the simultaneity (SJ) between the mouse press and the tone. The participants also rated their level of confidence in the AJ (1: least confident, 2: moderately confident, 3: very confident). The participant’s response was obtained *via* a dedicated response box. Assignment of the response key to the response box for the AJ and the SJ was counterbalanced across participants.

Throughout the experiment, the participants were instructed to not fluctuate their inter-tap interval (ITI) intentionally, press the mouse too hard, or stomp their foot to keep a tempo. They were also instructed that they should judge agency and simultaneity as independently as possible, as they are subjective judgments and may differ throughout the experiment.

The participants had a short and a long practice session before the main sessions to get accustomed to the tasks. The short practice session consisted of six trials of the listening phase of model tone sequence and the reproduction phase, in which the self-controlled and computer-controlled trials were alternated so that participants could recognize the difference between them. The long practice session consisted of 24 trials that were the same as the main session, except that there was a performance feedback (e.g., the stability of their tapping and whether their answer as to who controlled the tone output was correct or not). After completing all of the four main sessions, the participants were debriefed about the experiment.

### Data Analysis

Data from the practice sessions were not included in the analysis. Data from the test phase during the main sessions were analyzed. Irregular data, where one or more of the following criteria were not met, were screened out before the analysis: (1) there were no missing taps during computer-controlled trials, (2) the ratio of the ITI to the ISI of tones ranged from 0.8 to 1.25 during both self-controlled and computer-controlled trials, (3) the mean ITI ranged within ±100 ms from the ISI of tones, (4) the standard deviation of the ITI was below 100 ms, (5) the mean asynchrony between the onset of the participant’s tap and the onset of tone ranged from −200 to 40 ms, and (6) the standard deviation of the asynchrony was below 60 ms. These criteria were introduced based on previous research (Knoblich and Repp, 2009) with some modifications. In this screening process, 4.8 out of 96 trials (5.0%) were excluded per participant on average.

Asynchrony was defined as the time difference between the onset of a participant’s tap and the onset of tone and was negative if the tap preceded the tone. Mean asynchrony was calculated for each trial and each participant by averaging the asynchronies within a trial. The standard deviation (SD) of the asynchrony within a trial was also calculated for each trial and participant.

All statistical analyses were performed using the R version 3.6.1 environment (R Core Team, 2019). Non-linear fitting was conducted using the “nls” function in the “stats” package (R Core Team, 2019). The GLMM analysis was conducted using the “glmer” function in the “lmer4” package (Bates et al., 2015). The analysis of deviance was conducted using the “Anova” function in the “car” package (Fox and Weisberg, 2011). The pairwise comparisons between conditions were conducted by the “emmeans” function in the “emmeans” package (Lenth, 2016, 2018).

The analysis was conducted *via* the following steps. Firstly, the participants’ SJs between mouse presses and tones (the SJ data) were analyzed on a trial-by-trial basis to confirm whether the TWS changed after exposure to the delayed auditory feedback and whether the TR indeed occurred. Secondly, the participants’ AJs about who controlled the tones (the AJ data) were analyzed on a trial-by-trial basis to confirm whether the TWA changed through TR. Thirdly, the AJ data were analyzed on a participant-by-participant basis by the SDT framework to verify whether the modulation of the TWA was due to a change in the perceptual sensitivity or a change in the decision criterion. Finally, correlational analysis was conducted on the SJ and the AJ data to see how they were related.

#### Analysis of the Simultaneity Judgment Data

The data from the SJ task (the SJ data) were analyzed on a trial-by-trial basis. The data under the self-controlled trials and those under the computer-controlled trials were analyzed separately. The primary purpose of the analysis was to confirm whether the TWS between action and feedback was modulated after exposure to delayed feedback and whether TR indeed occurred. TR would manifest itself as a modulation of the psychometric function of the SJ against the mean asynchrony between the baseline and the delayed feedback conditions.

The secondary purpose of the analysis was to clarify how the participants used the sensorimotor cues in the SJ task. The most important sensorimotor cue would be a temporal asynchrony between the participant’s tap and the sensory feedback (Knoblich and Repp, 2009). In addition, as the participants experienced several pairings between tap and tone in the SJ task, a variability of the temporal asynchrony between the tap and the tone would be the sensorimotor cue as well.

The mean and SD of asynchrony were calculated for each computer-controlled trial, for each participant, for each feedback delay condition, and for each session. The first and second taps were not included in this analysis, as the feedback tones in these two taps were always self-controlled and were presented with an 80-ms delay.

First, a Gaussian function with variable height, width, and midpoint parameters was fitted to the SJ data against the mean asynchrony under the computer-controlled trials. The rationale of the Gaussian fitting is based on a characteristic of the SJ against the mean asynchrony: it peaks at a certain value of the mean asynchrony and gradually decreases thereafter. In addition, previous TR studies have typically used the Gaussian fitting as a function for the SJ against the mean asynchrony (e.g., Heron et al., 2009; Rohde et al., 2014a). One may argue that the Gaussian function is inappropriate, as the TWS between action and feedback is known to be asymmetrical (Franck et al., 2001; Sato and Yasuda, 2005; van Wassenhove et al., 2007; Rohde and Ernst, 2012; Farrer et al., 2013; Yarrow et al., 2013; Rohde et al., 2014b; Timm et al., 2014). However, the asymmetrical function is more difficult to fit to the observed data than the Gaussian. In addition, as there were too few data points at the extremely feedback-lagging side in the current data (e.g., <−150 ms of the mean asynchrony), there was no advantage of modeling these data by the asymmetrical function.

Each parameter of the Gaussian function was assumed to be different across each condition (baseline vs. delayed) and each session (first vs. second). Fitting was conducted by the *nls* function in the R environment.

$$S\,J_{i}\,\left(x\right)=a\,s\,y\,m_{i}\times e\,x\,p\,\left[\frac{-{\left(x-m\,u_{i}\right)}^{2}}{2\times s\,i\,g\,m\,a_{i}^{2}}\right]$$

where *SJ*_*i*_(*x*) is the response probability of the SJ (1 = synchronous, 0 = asynchronous); *x* is the mean asynchrony within a trial; and *asym*, *mu*, and *sigma* are the parameters of the Gaussian function representing height, midpoint, and width for each condition, respectively. The subscript *i* represents each condition and each session. Of note here is the fitting that was done for the SJ data of all participants. A popular way may be to do the fitting for each participant individually and then average the estimated functions across participants. However, individual fitting for each participant was difficult with the current data due to a small number of observations per participant (7–12 observations, average = 11.0).

Second, a half-Gaussian function with variable height and width was fitted to the SJ data against the SD of asynchrony under the computer-controlled trials. The half-Gaussian function was selected, as it is natural to assume that the mean rate of synchronous response decreases when the SD of asynchrony increases and also because the parameters were easy to interpret. Each parameter of the half-Gaussian function was assumed to be different across each condition (baseline vs. delayed) and each session (first vs. second). Fitting was conducted by the *nls* function in the R environment.

$$SJ_{i}\left(x\right)=asym_{i}\times exp\left[\frac{-x^{2}}{2\times s\,i\,g\,m\,a_{i}^{2}}\right]\left(x\geq 0\right)$$

where *SJ*_*i*_(*x*) is the response probability of the SJ (1 = synchronous, 0 = asynchronous); *x* is the SD of asynchrony within a trial; and *asym* and *sigma* are the parameters of the half-Gaussian function representing height and width for each condition, respectively. The subscript *i* represents each condition and each session.

The SJ data from the self-controlled trials were entered into the GLMM with a logit link function, with the feedback delay (baseline vs. delayed), session (first vs. second), and feedback delay × session interaction as fixed effects. As the random slope should be included in the model to ensure generality of the result (Barr et al., 2013; Magezi, 2015; Brauer and Curtin, 2018; Murayama, 2018), the model had a by-participant random intercept as well as a random slope for the feedback delay factor and for the session factor.

#### Analysis of the Agency Judgment Data

The data from the AJ task (AJ data) were analyzed on a trial-by-trial basis. As with the analysis of the SJ data, the AJ data under the self-controlled trials and the computer-controlled trials were analyzed separately. The modulation of the SoA after exposure to delayed feedback would manifest itself as a shift in the psychometric function of the AJ against the mean asynchrony.

First, a Gaussian function with variable height, width, and midpoint parameters was fitted to the AJ data against the mean asynchrony under the computer-controlled trials. As the first and the second taps were always self-controlled with a fixed delay (80 ms), they were not included in the analysis. Each parameter was assumed to be different across each condition (baseline vs. delayed) and each session (first vs. second).

$$A\,J_{i}\,\left(x\right)=a\,s\,y\,m_{i}\times e\,x\,p\,\left[\frac{-{\left(x-m\,u_{i}\right)}^{2}}{2\times s\,i\,g\,m\,a_{i}^{2}}\right]$$

where *AJ*_*i*_(*x*) is the response probability of the AJ (1 = self-controlled, 0 = computer-controlled); *x* is the mean asynchrony for each trial; and *asym*, *mu*, and *sigma* are the parameters of the Gaussian function representing height, midpoint, and width for the baseline and the delayed conditions, respectively. The subscript *i* represents each condition and each session.

Second, a half-Gaussian function with variable height and width was fitted to the AJ data against the SD of asynchrony under the computer-controlled trials. Each parameter of the half-Gaussian function was assumed to be different across each condition (baseline vs. delayed) and each session (first vs. second). Fitting was conducted by the *nls* function in the R environment.

$$AJ_{i}\left(x\right)=asym_{i}\times exp\left[\frac{-x^{2}}{2\times s\,i\,g\,m\,a_{i}^{2}}\right]\left(x\geq 0\right)$$

where *AJ*_*i*_(*x*) is the response probability of the AJ (1 = self-controlled, 0 = computer-controlled); *x* is the SD of asynchrony within a trial; and *asym* and *sigma* are the parameters of the half-Gaussian representing height and width for each condition, respectively. The subscript *i* represents each condition and each session.

As with the SJ data, the AJ data under the self-controlled trials were entered into the GLMM with a logit link function, with the feedback delay (baseline vs. delayed), the session (first vs. second), and the feedback delay × session interaction as fixed effects. The model had a by-participant random intercept as well as a random slope for the feedback delay factor and for the session factor.

The data from the AJ task (AJ data) were analyzed further on a participant-by-participant basis using the SDT framework. The purpose of the analysis was to clarify whether the change in the SoA after exposure to delayed auditory feedback was due to a change in perceptual sensitivity or decision criterion. The AJ data under both of the self-controlled trials and the computer-controlled trials were analyzed together.

The participants’ 2AFC judgments about the agency were categorized as a “hit” when they correctly judged that the auditory feedback was controlled by the computer in a computer-controlled trial and as a “false alarm” when they incorrectly judged that the auditory feedback was controlled by the computer in a self-controlled trial. The hit and false alarm rates were calculated for each condition, each session, and each participant. When the hit rate (or the false alarm rate) equaled 1.0, it was corrected by decreasing the number of “hit” (or “false alarm”) responses by 0.5. When the false alarm rate (or the hit rate) equaled 0.0, it was corrected by increasing the number of “false alarm” (or “hit”) responses by 0.5 (Wickens, 2002).

The subjective SoA ratings were defined as a combination of the AJ (self-controlled vs. computer-controlled) and the confidence rating about that judgment (least confident, moderately confident, or very confident). For example, if the participant’s judgment was “self-controlled” and their confidence rating was “very confident,” the SoA rating was defined as 6 (highest SoA), and if the participant’s judgment was “computer-controlled” and their confidence rating was “very confident,” the SoA rating was defined as 1 (lowest SoA). The other SoA ratings (2, 3, 4, 5) were defined in a similar manner. These six levels of SoA ratings were mobilized to determine the five levels of decision criterion in defining the receiver operating characteristic (ROC) curve with unequal variance under the noise and the signal.

Applying the SDT to the “hit” and “false alarm” rate with the SoA ratings, the sensitivity (*Az*) and the response bias (β) were obtained for each condition, each session, and each participant. The sensitivity (*Az*) corresponds to how well the participants discriminate about the agency. The response bias (β) corresponds to a tendency of responding toward either “self” or “computer” in the AJ task.

**FIGURE 3:**
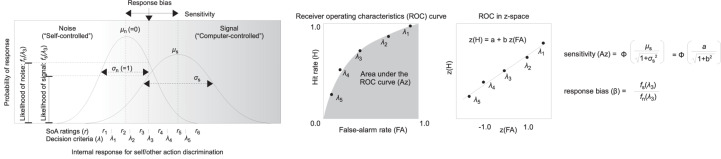
Graphical explanation of the application of the signal detection theory (SDT) to the agency judgment (AJ) data with sense of agency (SoA) ratings, assuming that the variance is unequal between the noise and the signal.

Figure 3 illustrates how the sensitivity (*Az*) and the response bias (β) are defined in the AJ task with confidence ratings (Swets and Pickett, 1982; Stanislaw and Todorov, 1999; Wickens, 2002). The horizontal axis in the figure represents a hypothetical internal response about the agency. The vertical axis in the figure represents a probability of the internal response. Each Gaussian curve corresponds to a probability of an internal response to the presence of noise (i.e., under the self-controlled trial) and to the presence of a signal (i.e., under the computer-controlled trial). The 2AFC-with-ratings model can handle situations where the variance of the noise distribution and the signal distribution is unequal (e.g., Wickens, 2002).

Sensitivity (*Az*) was defined as the area under the ROC curve that was calculated from the hit rate and the false alarm rate for each decision criterion, which were defined by the six levels of SoA ratings ranging from 1 (lowest SoA) to 6 (highest SoA) in the AJ task. The value of *Az* increases if the discrimination between the self-controlled and the computer-controlled trials becomes more accurate.

$$A\,z=\text{Φ}\,\left(\frac{\mu_{s}}{\sqrt{1+\sigma_{s}^{2}}}\right)=\text{Φ}\,\left(\frac{a}{\sqrt{1+b^{2}}}\right)$$

Here, Φ is a cumulative Gaussian function; μ_*s*_ is the center and $\sigma_{s}^{2}$ is the variance of the internal response distribution under the signal, which are equal to the intercept (*a*) and the slope (*b*) of the fitting line to the five data points of the ROC in a z-space (Figure 3, central and right panels).

The response bias (β) was defined as the ratio of the probability density at the middle of the decision criterion, which corresponds to the 2AFC judgment (self-controlled vs. computer-controlled) (i.e., λ_*3*_ in Figure 3). The value of β increases when the participants tend to respond that the auditory feedback is self-controlled.

$$\beta =\frac{f_{s}\,\left(\lambda_{3}\right)}{f_{n}\,\left(\lambda_{3}\right)}$$

where *f*_*s*_ and *f*_*n*_ are Gaussian functions under the signal and under the noise, respectively. λ_*3*_ is the middle of the decision criteria, which corresponds to the AJ (Figure 3, left panel).

## Results

### Simultaneity Judgment Data From the Computer-Controlled Trials

**FIGURE 4:**
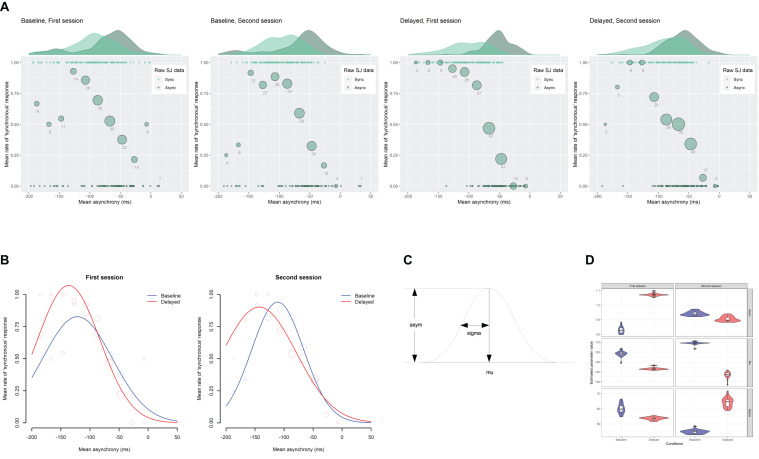
Results from the analysis on a trial-by-trial basis about the simultaneity judgment (SJ) data under the computer-controlled trials against the mean asynchrony. **(A)** Scatter plots of the raw SJ data against the mean asynchrony that is pooled across participants for each condition in two sessions. The probability density of the data is displayed as a curve outside the plot. The mean rate of the “synchronous” responses against the 12 bins of the mean asynchrony is displayed as a circle within the plot. The size of the circle reflects the number of observations in the bin, which is also indicated by the digit below the circle. **(B)** Fitted Gaussian functions of the SJ against the mean asynchrony for each condition in two sessions. The mean rate of the “synchronous” response against the binned mean asynchrony was also displayed for reference. The size of the circle reflects the number of observations. **(C)** The asym, mu, and sigma are the parameters of Gaussian representing height, midpoint, and width. **(D)** Violin plots with boxplots representing the distribution of the estimated parameters from the 18 jackknife replications of the fitting of the Gaussian function to the SJ against the mean asynchrony.

Figure 4A shows the scatter plots of the raw SJ data against the mean asynchrony under the computer-controlled trials for each condition in two sessions, which is pooled across participants. The probability density of the data is displayed as a curve outside the plot. The mean rate of the “synchronous” responses against the 12 bins of the mean asynchrony is displayed as a circle, whose size reflects the number of the observations in the bin. The midpoints of the bin are set to −187, −167, −147, −127, −107, −87, −67, −47, −27, −7, 13, and 33 ms.

Figure 4B demonstrates the fitted Gaussian functions to the SJ against the mean asynchrony under the computer-controlled trials for each condition and each session. The mean rate of “synchronous” responses against the binned mean asynchrony is also displayed for reference. The size of the circle reflects the number of observations. The estimated values of the parameters (Figure 4C) are shown in Table 1. The goodness of fit of the model was evaluated by Pearson product-moment correlation squared (*r*^2^) between the observed mean rate of “synchronous” responses and the predicted rate of “synchronous” responses by the model at the 12 bins of the mean asynchrony. The correlation was weighted using the number of observations in each bin (Bland and Altman, 1995). The *r*^2^ values of the model are shown in Table 1. As shown in the figure, the mean rate of “synchronous” responses was systematically varied against the mean asynchrony, indicating that participants used the mean asynchrony as a sensorimotor cue in the SJ.

**TABLE 1:** Estimated parameter values of the fitted psychometric curve of the mean rate of “synchronous” response against the mean asynchrony.

	asym				mu				sigma				Goodness of fit (*r*^2^)
	Mean	SE	LCI	UCI	Mean	SE	LCI	UCI	Mean	SE	LCI	UCI	
**First session**													
Baseline (80 ms)	0.83	(0.06)	0.72	0.95	−121.6	(6.2)	−143.6	−108.2	60.1	(7.2)	44.0	90.3	0.76
Delayed (180 ms)	1.07	(0.06)	1.00	1.13	−136.7	(7.0)	−144.7	−125.3	53.7	(5.7)	45.7	60.8	0.95
Delayed – Baseline	0.24**				−15.1				−6.4				
**Second session**													
Baseline (80 ms)	0.94	(0.05)	0.86	1.03	−111.0	(4.0)	−119.8	−103.8	44.8	(4.3)	38.0	54.7	0.93
Delayed (180 ms)	0.90	(0.08)	0.79	1.14	−142.8	(13.5)	−206.2	−126.0	63.9	(10.5)	49.7	101.8	0.90
Delayed – Baseline	−0.04				−31.8**				19.1*				
**Second session – First session**													
Baseline (80 ms)	0.11				10.6				−15.3				
Delayed (180 ms)	−0.17				−6.1				10.2				
Delayed – Baseline	−0.28*				−16.7				25.5*				

The goodness of fit of the model is evaluated by Pearson product-moment correlation squared (*r*^2^) between observed and predicted rates. SE, standard error of mean; LCI/UCI, lower/upper 95% bootstrap percentile CI. *p < 0.05, **p < 0.01. Statistical tests were done by 95% bootstrap percentile CIs.

To test formally if the estimated parameter values were significantly different among the conditions, 95% bootstrap percentile confidence intervals (CIs) were calculated for each parameter (Efron, 1979; Freedman, 1981). Bootstrap distributions of each parameter were obtained after 2,000 simulations with replacement. Then, multiple (two-tailed) comparisons between the four conditions (feedback delay × session) were executed using equal-tail bootstrap *p* values (MacKinnon, 2006, 2007) with no adjustment (Rothman, 1990).

The multiple comparisons revealed that the mu (the midpoint of Gaussian) was significantly different between the baseline and the delayed conditions in the second session (−111.0 vs. −142.8 ms, *p* = 0.003) but not in the first session (−121.6 vs. −136.7 ms, *p* = 0.181), indicating that the center of the TWS shifted in the direction of the delay by 31.8 ms from the baseline to the delayed feedback condition in the second session. The asym (the height of Gaussian) was significantly larger by 0.24 for the baseline (0.83) than the delayed (1.07) condition in the first session (*p* = 0.001) but not in the second session (0.94 vs. 0.90, *p* = 0.824). The sigma (the width of Gaussian) was also significantly different between the baseline and the delayed conditions in the second session (44.8 vs. 63.9 ms, *p* = 0.035) but not in the first session (60.1 vs. 53.7 ms, *p* = 0.499), indicating that the width of the TWS was widened by 19.1 ms from the baseline to the delayed feedback condition in the second session.

One may suspect that these estimated parameter values might have been influenced by the data of a few participants. To check this, a grouped (stratified) jackknife method (Quenouille, 1949, 1956; Tukey, 1958; Efron, 1980; Kott, 2001) was applied to the data, in which the data were split into subsets of each participant and the non-linear Gaussian fitting were repeatedly executed on the new data in which one of the participants was removed. The distribution of the estimated parameters from the 18 jackknife replications is shown as violin plots with boxplots (Figure 4D), in which the probability density, median, and interquartile range of the data are shown. The upper or lower whisker extends from the first or the third quartile to the largest or smallest value no further than 1.5 times as long as the interquartile range from the quartiles. The black dots represent outliers, which were beyond the end of the whiskers. The figure indicates that all of the parameters showing a significant difference between the baseline and the delayed conditions—the asym in the first session (top left panel), the mu in the second session (middle right panel), and the sigma in the second session (bottom right panel)—seemed to be different enough between the baseline and the delayed conditions, although there were few influential participants (black dots in the figure).

The modulation of the midpoint (mu) and the width (sigma) of the TWS after exposure to the delay make sense. The shift of the midpoint of the TWS in the direction of the delay would represent a shift of the point of subjective simultaneity (PSS), and the widening of the width of the TWS would represent a loss of sensitivity of the simultaneity [i.e., just-noticeable difference (JND)], which can be interpreted as a compensation of prediction error caused by the delay of the sensory feedback. The modulation of the height (asym) of the TWS might reflect the process of modulation of the TWS; that is, the midpoint shifted first, then the width widened.

**FIGURE 5:**
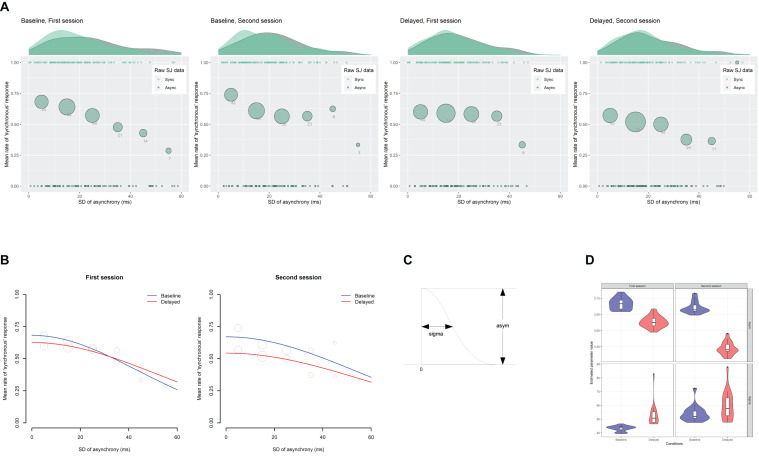
Results from the analysis on a trial-by-trial basis about the simultaneity judgment (SJ) data under the computer-controlled trials against the SD of asynchrony. **(A)** Scatter plots of the raw SJ data against the SD of asynchrony that is pooled across participants for each condition in two sessions. The probability density of the data is displayed as a curve outside the plot. The mean rate of the “synchronous” responses against the six bins of the SD of asynchrony is displayed as a circle within the plot. The size of the circle reflects the number of observations in the bin, which is also indicated by the digit below the circle. **(B)** Fitted half-Gaussian functions of the SJ against the SD of asynchrony for each condition in two sessions. Mean rate of “synchronous” response against the binned SD of asynchrony was also displayed for reference. The size of the circle reflects the number of observations. **(C)** The asym and sigma are the parameters of the half-Gaussian representing height and width. **(D)** Violin plots with boxplots representing the distribution of the estimated parameters from the 18 jackknife replications of the fitting of the half-Gaussian function to the SJ against the SD of asynchrony.

Figure 5A indicates the scatter plots of the raw SJ data against the SD of asynchrony under the computer-controlled trials for each condition in two sessions, which is pooled across participants. The probability density of the data and the mean rate of the “synchronous” responses against the six bins of the SD of asynchrony are also displayed. The midpoints of the bin are set to 5, 15, 25, 35, 45, and 55 ms. Figure 5B shows the fitted half-Gaussian functions to the SJ against the SD of asynchrony for each condition and each session. The mean rate of the “synchronous” response against the binned SD of asynchrony is also displayed for reference. The size of the circle reflects the number of observations. Estimated values of parameters (Figure 5C) are shown in Table 2. The goodness of fit of the model was evaluated by Pearson product-moment correlation squared (*r*^2^) between the observed mean rate of the “synchronous” responses and the predicted rate of “synchronous” responses by the model at the six bins of the SD of asynchrony. The correlation was weighted using the number of observations in each bin (Bland and Altman, 1995). The *r*^2^ values of the model are shown in Table 2. As shown in the figure, the mean rate of synchronous response was systematically varied against the SD of asynchrony, indicating that participants used the fluctuation of asynchrony as a sensorimotor cue for the SJ.

**TABLE 2:** Estimated parameter values of the fitted linear regression of the mean rate of “synchronous” response against the SD of asynchrony.

	asym				sigma				Goodness of fit (*r*^2^)
	Mean	SE	LCI	UCI	Mean	SE	LCI	UCI	
**First session**									
Baseline (80 ms)	0.68	(0.05)	0.58	0.78	43.1	(9.2)	30.5	83.8	0.99
Delayed (180 ms)	0.63	(0.05)	0.54	0.74	51.7	(19.6)	31.0	164.5	0.59
Delayed – Baseline	−0.05				8.6				
**Second session**									
Baseline (80 ms)	0.67	(0.05)	0.59	0.78	53.3	(18.2)	32.1	163.4	0.47
Delayed (180 ms)	0.54	(0.05)	0.47	0.66	57.8	(27.2)	30.6	198.3	0.20
Delayed – Baseline	−0.13				4.5				
**Second session – First session**									
Baseline (80 ms)	−0.01				10.20				
Delayed (180 ms)	−0.09				6.10				
Delayed – Baseline	−0.08				−4.10				

*The goodness of fit of the model is evaluated by Pearson product-moment correlation squared (*r*^2^) between observed and predicted rates. SE, standard error of mean; LCI/UCI, lower/upper 95% bootstrap percentile CI.*

The difference in the estimated parameter values between the baseline and the delayed conditions was statistically tested using 95% bootstrap percentile CIs and equal-tail bootstrap *p* values. Multiple comparisons with no adjustment of *p* values revealed that the asym (the height of half-Gaussian) in the second session approached significance but was not significantly smaller (*p* = 0.078) for the delayed (0.54) than the baseline condition (0.67), suggesting that the participants tended to judge less simultaneity for zero fluctuation of asynchrony in the second session under the delayed condition than the baseline condition. The sigma (the width of half-Gaussian) was not significantly different between the baseline and the delayed conditions in both sessions (*p* > 0.05), suggesting that the TR did not modulate the participants’ sensitivity to the fluctuation of asynchrony when they judged simultaneity. However, the SEs were so large in the estimation of the sigma (9.2∼27.2 ms; Table 2) that we cannot claim it strongly. In fact, the sigma tended to be larger for the delayed than the baseline condition in the first (43.1 vs. 51.7 ms) and the second sessions (53.3 vs. 57.8 ms), which may suggest that sensitivity to the fluctuation of asynchrony was decreased by the TR.

To check whether a few influential participants affected the fitting result, the jackknife procedure was executed on the half-Gaussian fitting of the SJ data against the SD of asynchrony. The distribution of the estimated parameters from the 18 jackknife replications to the new dataset, in which one of the participants was removed, is shown as violin plots with boxplots (Figure 5D). The jackknife results demonstrate that there seemed to be no difference between the baseline and the delayed condition with the asym and the sigma parameters, although there were a few influential participants (black dots in the figure). It must be noted that the asym in the second session (top right panel) could be different between the baseline and the delayed conditions, though the statistical test using 95% bootstrap percentile CIs in the previous section did not show a significant difference.

### Simultaneity Judgment Data From the Self-Controlled Trials

**FIGURE 6:**
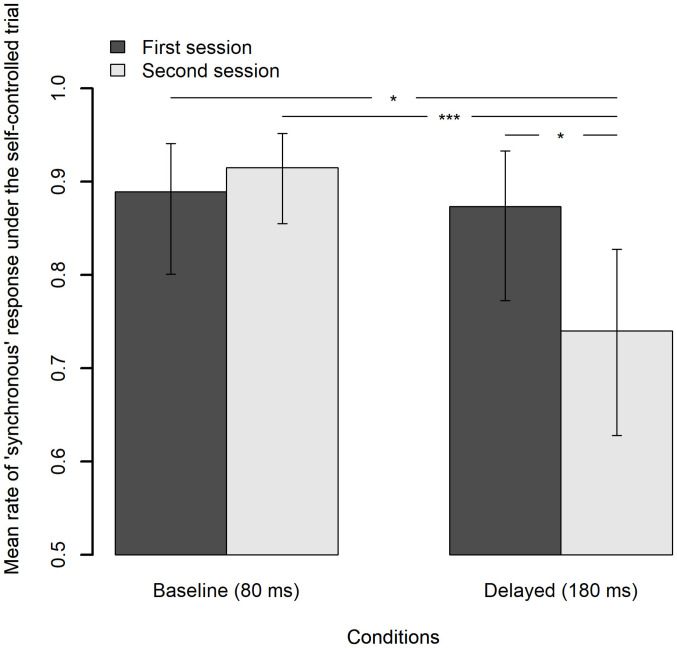
Mean rate of “synchronous” response under the self-controlled trials for each feedback delay and each session, in which the feedback delay is fixed to 80 ms. The error bar represents 95% confidence interval of the mean. *p < 0.05, ***p < 0.001.

Figure 6 shows the mean rate of “synchronous” response under the self-controlled trials for each feedback delay and each session with a 95% CI. Analysis of deviance revealed that the main effect of the feedback delay and the interaction between the feedback delay and the session were significant [χ^2^(1) = 7.6, *p* = 0.006; χ^2^(1) = 8.7, *p* = 0.003, respectively]. The effect of session approached significance but was not significant, χ^2^(1)= 3.0, *p* = 0.085. Pairwise comparisons using Tukey’s honestly significant difference (HSD) method revealed that the mean rate of “synchronous” response under the delayed feedback condition in the second session decreased by 13.3% from that in the first session, *z* = 3.01, *p* = 0.014, and was lower than that under the baseline condition in the second session by 17.5%, *z* = 3.99, *p* < 0.001, as well as in the first session by 14.9%, *z* = 2.99, *p* = 0.015. Meanwhile, the difference in the mean rate of “synchronous” response under the baseline condition between the first session and the second session (−2.6%) was not significant, *z* = −0.84, *p* = 0.835. The results clearly indicate that the TR actually occurred after exposure to delayed auditory feedback.

### Agency Judgment Data From the Computer-Controlled Trials

**FIGURE 7:**
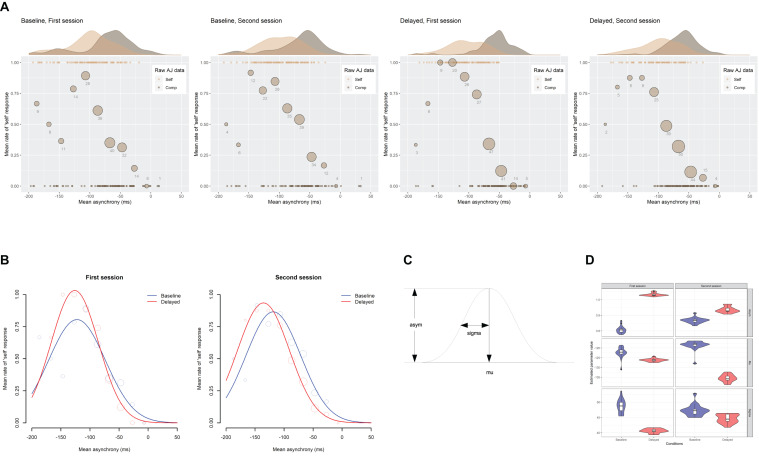
Results from the analysis on a trial-by-trial basis about the agency judgment (AJ) data under the computer-controlled trials against the mean asynchrony. **(A)** Scatter plots of the raw AJ data against the mean asynchrony that is pooled across participants for each condition in two sessions. The probability density of the data is displayed as a curve outside the plot. The mean rate of the “self” responses against the 12 bins of the mean asynchrony is displayed as a circle within the plot. Size of the circle reflects the number of observations in the bin, which is also indicated by the digit below the circle. **(B)** Fitted Gaussian functions of the AJ against the mean asynchrony for each condition in two sessions. Mean rate of “self” response against the binned mean asynchrony was also displayed for reference. The size of the circle reflects the number of observations. **(C)** The asym, mu, and sigma are the parameters of Gaussian representing height, midpoint, and width. **(D)** Violin plots with boxplots representing the distribution of the estimated parameters from the 18 jackknife replications of the fitting of the Gaussian function to the AJ against the mean asynchrony.

Figure 7A indicates the scatter plots of the raw AJ data against the mean asynchrony under the computer-controlled trials for each condition in two sessions, which is pooled across participants. The probability density of the data and the mean rate of the “self” responses against the 12 bins of the mean asynchrony are also displayed. Figure 7B shows the fitted Gaussian functions to the AJ against the mean asynchrony under the computer-controlled trials for each condition and each session. The mean rate of “self” response against the binned mean asynchrony is also displayed for reference. The size of the circle reflects the number of observations. The estimated values of the parameters (Figure 7C) are shown in Table 3. The goodness of fit of the model (*r*^2^) was evaluated in the same way as the SJ data. As shown in the figure, the mean rate of “self” response was systematically varied against the mean asynchrony, indicating that participants used the asynchrony as a sensorimotor cue for the AJ.

**TABLE 3:** Estimated parameter values of the fitted psychometric curve of the mean rate of “self” response against the mean asynchrony.

	asym				mu				sigma				Goodness of fit (*r*^2^)
	Mean	SE	LCI	UCI	Mean	SE	LCI	UCI	Mean	SE	LCI	UCI	
**First session**													
Baseline (80 ms)	0.81	(0.06)	0.70	0.94	−122.4	(4.9)	−140.5	−108.5	48.8	(5.2)	32.1	67.1	0.74
Delayed (180 ms)	1.03	(0.06)	0.97	1.10	−126.2	(4.2)	−136.3	−117.6	40.7	(3.6)	34.8	47.8	0.98
Delayed – Baseline	0.22**				−3.8				−8.1				
**Second session**													
Baseline (80 ms)	0.87	(0.05)	0.77	0.97	−118.6	(5.0)	−134.1	−109.3	47.2	(5.0)	38.8	61.5	0.89
Delayed (180 ms)	0.94	(0.08)	0.80	1.08	−135.6	(6.8)	−152.8	−122.4	44.7	(5.3)	34.8	57.2	0.99
Delayed – Baseline	0.07				−17.0				−2.5				
**Second session – First session**													
Baseline (80 ms)	0.06				3.8				−1.6				
Delayed (180 ms)	−0.09				−9.4				4.0				
Delayed – Baseline	−0.15				−13.2				5.6				

The goodness of fit of the model is evaluated by Pearson product-moment correlation squared (*r*^2^) between observed and predicted rates. SE, standard error of mean; LCI/UCI, lower/upper 95% bootstrap percentile CI. **p < 0.01. Statistical tests were done by 95% bootstrap percentile CIs.

After calculating 95% bootstrap percentile CIs for each parameter, multiple comparisons (two-tailed) between conditions were executed using equal-tail bootstrap *p* values with no adjustment.

The multiple comparisons revealed that the asym (the height of Gaussian) was significantly larger by 0.22 in the baseline (0.81) than the delayed (1.03) condition in the first session (*p* = 0.001) but not in the second session (0.87 vs. 0.94, *p* = 0.424). The mu (the midpoint of Gaussian) in the second session approached significance but was not significantly different between the baseline and the delayed conditions (−118.6 vs. −135.6 ms, *p* = 0.080), suggesting that the center of the TWA tended to shift in the direction of the delay by 17.0 ms from the baseline to the delayed feedback condition in the second session. The sigma (the width of Gaussian) was not significantly different between the baseline and the delayed conditions in both sessions (*p* > 0.05).

To check if there were a few influential participants affecting the fitting result, the jackknife procedure was applied to the Gaussian fitting of the AJ data against the mean asynchrony (Figure 7D). The figure indicates that the parameter showing a significant difference between the baseline and the delayed condition—the asym in the first session (top left panel)—seemed to be different enough between the baseline and the delayed conditions, although there were few influential participants (black dots in the figure). It is worth noting here that the mu in the second session (middle right panel) seemed to be different between the baseline and the delayed conditions. However, if the influential participants’ data were removed (black dots in the figure), the difference becomes smaller. The sigma in the first session (bottom left panel) also seemed to be different between the baseline and the delayed conditions, although the statistical test above did not show a significant difference.

**FIGURE 8:**
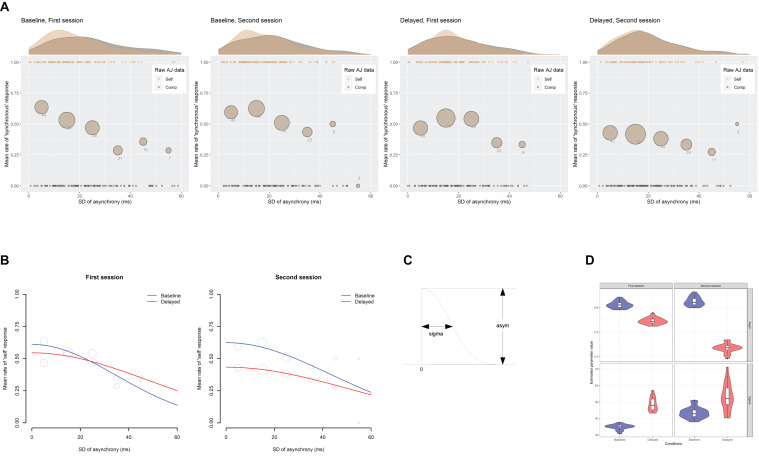
Results from the analysis on a trial-by-trial basis about the agency judgment (AJ) data under the computer-controlled trials against the SD of asynchrony. **(A)** Scatter plots of the raw AJ data against the SD of asynchrony that is pooled across participants for each condition in two sessions. The probability density of the data is displayed as a curve outside the plot. The mean rate of the “self” responses against the six bins of the SD of asynchrony is displayed as a circle within the plot. The size of the circle reflects the number of observations in the bin, which is also indicated by the digit below the circle. **(B)** Fitted half-Gaussian functions of the AJ against the SD of asynchrony for each condition in two sessions. Mean rate of “self” response against the binned SD of asynchrony was also displayed for reference. The size of the circle reflects the number of observations. **(C)** The asym and sigma are the parameters of the half-Gaussian representing height and width. **(D)** Violin plots with boxplots representing the distribution of the estimated parameters from the 18 jackknife replications of the fitting of the half-Gaussian function to the AJ against the SD of asynchrony.

Figure 8A indicates the scatter plots of the raw AJ data against the SD of asynchrony under the computer-controlled trials for each condition in two sessions, which is pooled across participants. The probability density of the data and the mean rate of the “self” responses against the six bins of the SD of asynchrony are also displayed. Figure 8B shows the fitted half-Gaussian functions to the AJ against the SD of asynchrony for each condition and each session. The mean rate of “self” response against the binned SD of asynchrony is also displayed for reference. The size of the circle reflects the number of observations. The estimated values of the parameters (Figure 8C) are shown in Table 4. The goodness of fit of the model (*r*^2^) was evaluated in the same way as the SJ data. As shown in the figure, the mean rate of “self” response was systematically varied against the SD of asynchrony, indicating that participants used the fluctuation of asynchrony as a sensorimotor cue in the AJ.

**TABLE 4:** Estimated parameter values of the fitted linear regression of the mean rate of “self” response against the SD of asynchrony.

	asym				sigma				Goodness of fit (*r*^2^)
	Mean	SE	LCI	UCI	Mean	SE	LCI	UCI	
**First session**									
Baseline (80 ms)	0.61	(0.05)	0.51	0.73	34.8	(6.8)	23.9	60.1	0.84
Delayed (180 ms)	0.55	(0.05)	0.46	0.66	48.2	(18.9)	29.5	159.2	0.41
Delayed – Baseline	−0.06				13.4				
**Second session**									
Baseline (80 ms)	0.63	(0.05)	0.53	0.73	43.1	(11.8)	28.6	104.3	0.71
Delayed (180 ms)	0.43	(0.05)	0.36	0.55	51.2	(25.3)	25.9	176.1	0.64
Delayed – Baseline	−0.20*				8.1				
**Second session – First session**									
Baseline (80 ms)	0.02				8.30				
Delayed (180 ms)	−0.12				3.00				
Delayed – Baseline	−0.14				−5.30				

The goodness of fit of the model is evaluated by Pearson product-moment correlation squared (*r*^2^) between observed and predicted rates. SE, standard error of mean; LCI/UCI, lower/upper 95% bootstrap percentile CI. *p < 0.05. Statistical tests were done by 95% bootstrap percentile CIs.

The difference in the estimated parameter values between the baseline and the delayed conditions was statistically tested using 95% bootstrap percentile CIs and equal-tail bootstrap *p* values. Multiple comparisons with no adjustment of *p* values revealed that the asym (the height of Gaussian) was significantly smaller by 0.20 for the delayed (0.43) than the baseline (0.63) condition in the second session (*p* = 0.016), indicating that the participants judged less agency for zero fluctuation of asynchrony in the second session under the delayed condition compared with the baseline. The sigma (the width of half-Gaussian) was not significantly different between the baseline and the delayed conditions in both sessions (*p* > 0.05), suggesting that the TR did not modulate the participants’ sensitivity to the fluctuation of asynchrony when they judged the agency. However, the SEs were so large in the estimation of the sigma (6.8∼25.3 ms; Table 4) that we cannot claim it strongly. In fact, the sigma tended to be larger for the delayed than the baseline condition in the first (34.8 vs. 48.2 ms) and the second session (43.1 vs. 51.2 ms), which may suggest that the sensitivity to the fluctuation of asynchrony was decreased by the TR.

To check whether a few influential participants affected the fitting result, the jackknife procedure was applied to the half-Gaussian fitting of the AJ data against the SD of asynchrony. The jackknife results are shown in Figure 8D, indicating that the parameters showing a significant difference between the baseline and the delayed conditions—the asym in the second session (top right panel)—seemed to be different enough between the two conditions. It is worth noting that the sigma in the first session (bottom left panel) could be different between the baseline and the delayed condition, though the statistical test using 95% bootstrap percentile CIs in the previous section did not show a significant difference.

### Agency Judgment Data From the Self-Controlled Trials

**FIGURE 9:**
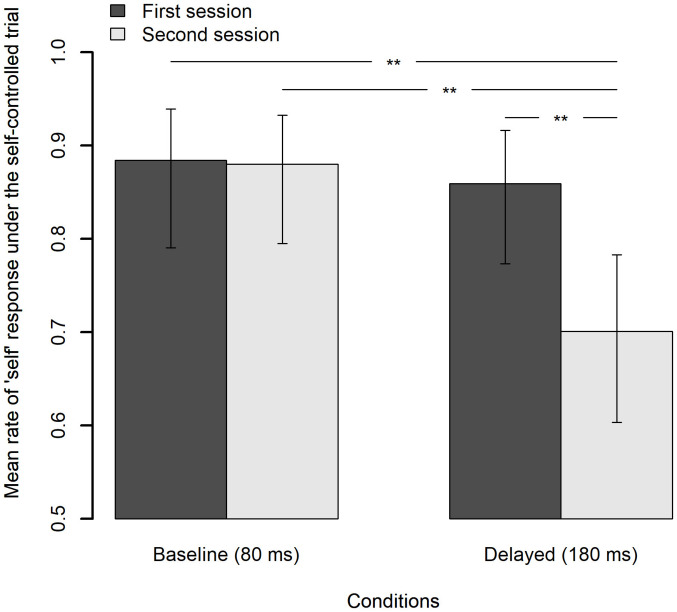
Mean rate of “self” response under the self-controlled trials for each feedback delay and each session, in which the feedback delay is fixed to 80 ms. The error bar represents a 95% confidence interval of the mean. **p < 0.01.

Figure 9 shows the mean rate of “self” response under the self-controlled trials for each feedback delay and each session with a 95% CI. Analysis of deviance revealed that the effects of the feedback delay, session, and interaction feedback delay and session were all significant [χ^2^(1) = 6.58, *p* = 0.010; χ^2^(1) = 5.08, *p* = 0.024; χ^2^(1) = 5.92, *p* = 0.015, respectively]. Pairwise comparisons using Tukey’s HSD method revealed that the mean rate of “self” response under the delayed feedback condition in the second session significantly decreased by 15.8% from that in the first session, *z* = 3.22, *p* = 0.007, and was also significantly lower than that under the baseline condition in the second session by 17.9%, *z* = 3.46, *p* = 0.003, as well as in the first session by 18.3%, *z* = 3.20, *p* = 0.008. Meanwhile, the difference of the mean rate of “self” response under the baseline condition between the first and the second session (0.4%) was not significant, *z* = 0.13, *p* = 0.999. The results clearly indicate that the decrement in the SoA actually occurred after exposure to delayed auditory feedback.

### Results From the Signal Detection Theory Analysis on the Agency Judgment Data

The observed data show that the mean estimated variance of signal distribution (σ_*s*_) for all participants, conditions, and sessions was 1.28 (95% CI = 1.20–1.37), which was significantly larger than the hypothetical variance of the noise distribution (σ_*n*_ = 1), *t*(71) = 7.99, *p* < 0.001. As the distribution of the σ_*s*_ was not normal (Shapiro–Wilk normality test, *W* = 0.84, *p* < 0.001), their inverse values (1/σ_*s*_), which were normally distributed (Shapiro–Wilk normality test, *W* = 0.99, *p* = 0.900), were averaged and inverted again to obtain the mean value of the σ_*s*_. The 95% CIs were obtained in the same way. The *t*-test was also executed on the 1/σ_*s*_. The larger σ_*s*_ over the σ_*n*_ indicated that it is valid to adopt the unequal variance model in the SDT.

**FIGURE 10:**
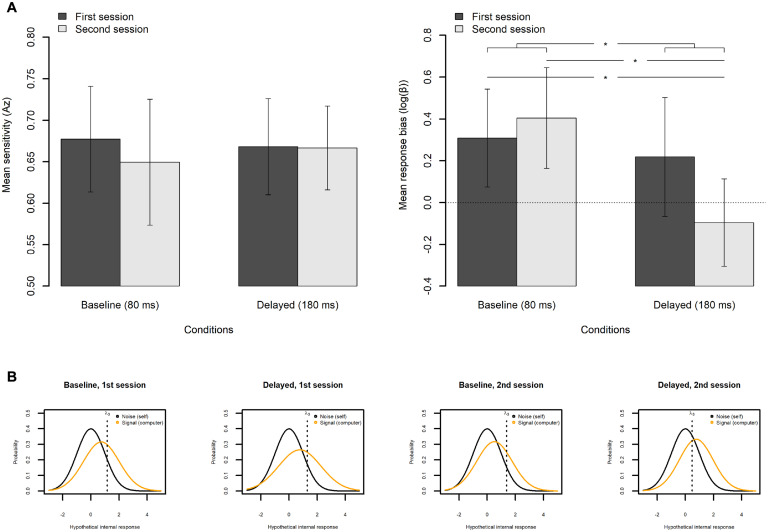
Results from the signal detection theory (SDT) analysis on the agency judgment (AJ) data. **(A)** Mean sensitivity (*Az*) and response bias (β) of the AJ in each condition in each session. The error bar represents a 95% confidence interval of the mean. *p < 0.05. **(B)** Estimated response probability function within the SDT framework. Median was used as a representative value of each parameter.

Figure 10A shows the mean sensitivity (*Az*) and the mean log(β) for each feedback delay and each session with a 95% CI. The values of the sensitivity (*Az*) were entered into the linear mixed-effects model, with the feedback delay (baseline vs. delayed), session (first vs. second), and feedback delay × session interaction as fixed effects. The model had by-participant random slopes for the feedback delay and the session and had by-participant one random intercept. Analysis of deviance revealed that none of the fixed effects were significant [the effect of the feedback delay: χ^2^(1) = 0.02, *p* = 0.893; the effect of the session: χ^2^(1) = 0.30, *p* = 0.587; and the interaction between feedback delay and session: χ^2^(1) = 0.39, *p* = 0.532]. The results indicate that an exposure to delayed auditory feedback did not change the perceptual sensitivity of agency.

As the response bias measure β is a ratio, it is common to analyze the natural logarithm of β [log(β)] (Stanislaw and Todorov, 1999). The values of log(β) were entered into the linear mixed-effects model, with the feedback delay (baseline vs. delayed), session (first vs. second), and feedback delay × session interaction as fixed effects. The model had by-participant random slopes for the feedback delay and the session and had by-participant one random intercept. Analysis of deviance revealed that the effects of the feedback delay, χ^2^(1) = 5.96, *p* = 0.015, and the interaction between the feedback delay and the session, χ^2^(1) = 6.32, *p* = 0.012, were significant. Pairwise comparisons using Tukey’s HSD method revealed that the mean log(β) under the delayed feedback condition in the second session was significantly lower by 0.50 from the baseline condition in the second session, *t*(29.8) = 3.43, *p* = 0.009, and was significantly lower by 0.40 from the baseline condition in the first session, *t*(17.0) = 2.99, *p* = 0.037. However, the difference between the first and the second session under the delayed condition was not significant, *t*(31.0) = 2.26, *p* = 0.130. These results indicate that exposure to the delayed auditory feedback shifted the response bias (β) to decrease the number of responses that were judged as self-controlled, suggesting that the participants became more “conservative” in judging the agency in this condition.

It is worth noting here that the mean values of the log(β) were above zero in both sessions under the baseline condition and in the first session under the delayed condition. As the zero of the log(β) means the participants judged the auditory feedback as self-controlled and computer-controlled equally, the positive bias clearly indicates a general tendency to attribute the sensory feedback to a self-controlled outcome (Knoblich and Repp, 2009).

Figure 10B shows the estimated response probability function within the SDT framework for reference. As the estimated parameters characterizing each distribution (i.e., μ_*s*_, σ_*s*_) and the decision criterion (i.e., λ_*3*_) were not normally distributed, the median was adopted as a representative value for each parameter. As is clearly seen, the relative locations of the two probability functions (i.e., μ) in the presence of noise (i.e., self-controlled) and in the presence of signal (i.e., computer-controlled) almost remained the same across conditions and sessions. However, the decision criterion (λ_*3*_) shifted only under the delayed condition in the second session (rightmost panel), which corresponds to the lowered mean log(β) under the delayed feedback condition in the second session in Figure 10A.

It is also worth noting here that the width of the signal distribution (i.e., computer-controlled trials) under the delayed condition in the first session (middle left panel) was slightly larger than that of the other distributions, which might suggest that the participants became somewhat uncertain about what the computer-controlled trials sounded like in this condition.

### Relationship Between Simultaneity Judgment and Agency Judgment

One may suspect that the participants might judge SJ and AJ identically, as they were both performed within the same trial. In this section, SJ and AJ are compared with each other to see if the participants judged synchrony and agency in a similar manner or not.

**FIGURE 11:**
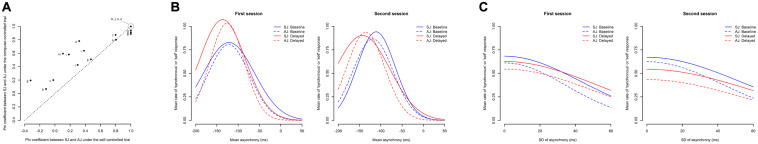
Relationship between the simultaneity judgment (SJ) and the agency judgment (AJ) data. **(A)** Correlation between the SJ and AJ for each participant (phi coefficients). The *x* axis represents the phi coefficients under the self-controlled trials, while the *y* axis represents those under the computer-controlled trials. Each alphabetical letter indicates an individual participant. **(B)** Overlapped fitted Gaussian function of the SJ (Figure 4B) and AJ (Figure 7B) against the mean asynchrony. **(C)** Overlapped fitted half-Gaussian function of the SJ (Figure 5B) and AJ (Figure 8B) against the SD of asynchrony.

First, the correlations between SJ and AJ were calculated on a trial-by-trial basis for each participant. Phi coefficients between SJ (1 = synchronous vs. 0 = not synchronous) and AJ (1 = self-controlled vs. 0 = computer-controlled) were calculated for each participant for the computer-controlled and self-controlled trials separately. Figure 11A shows a scatter plot between the phi coefficients under the self-controlled trials (*x* axis) and those under the computer-controlled trials (*y* axis). Each alphabetical letter indicates an individual participant. If the participants judged SJ and AJ in exactly the same way, the phi coefficient would be 1.0 for both self-controlled and computer-controlled trials. Thus, data would be concentrated in the top-right corner of the figure. As can be seen in the figure, seven out of the 18 participants (indicated as “d,” “e,” “h,” “j,” “n,” “o,” and “q”) are concentrated in the top-right corner of the figure, but the other 11 participants are not. This means that the majority of the participants (11 out of 18) judged that SJ and AJ were different from each other.

Second, the fitted Gaussian function of the mean rate of the “synchronous” response against the mean asynchrony was compared with that of the mean rate of the “self” response against the mean asynchrony. To verify if the shape of the estimated function differed between them, multiple (two-tailed) comparisons between SJ and AJ were performed for each parameter using equal-tail bootstrap *p* values with no adjustment. Figure 11B shows an overlaid figure of the fitted Gaussian function of the mean rate of the “synchronous” response (Figure 4B) and the mean rate of the “self” response (Figure 7B) against the mean asynchrony without the reference data points.

The multiple comparisons revealed that the sigma (the width of Gaussian) was significantly larger for SJ than AJ under the delayed condition in both the first session (SJ: 53.7 ms vs. AJ: 40.7 ms, *p* = 0.015) and the second session (SJ: 63.9 ms vs. AJ: 44.7 ms, *p* = 0.020), while they were not significantly different under the baseline condition in both the first session (SJ: 60.1 ms vs. AJ: 48.8 ms, *p* = 0.208) and the second session (SJ: 44.8 ms vs. AJ: 47.2 ms, *p* = 0.628). On the other hand, the mu (the midpoint of Gaussian) was not significantly different between SJ and AJ under the delayed condition in both the first session (SJ: −136.7 ms vs. AJ: −126.2 ms, *p* = 0.143) and the second session (SJ: −142.8 ms vs. AJ: −135.6 ms, *p* = 0.353) nor under the baseline condition in both the first session (SJ: −121.6 ms vs. AJ: −122.4 ms, *p* = 0.941) and the second session (SJ: −111.0 ms vs. AJ: −118.6 ms, *p* = 0.120). Likewise, the asym (the height of Gaussian) was not significantly different between SJ and AJ under both conditions in both sessions (all *p*s > 0.05).

Finally, the fitted half-Gaussian function of the mean rate of the “synchronous” response against the SD of asynchrony was compared with that of the mean rate of the “self” response against the SD of asynchrony. The multiple (two-tailed) comparisons between conditions were executed for each parameter using equal-tail bootstrap *p* values with no adjustment. Figure 11C shows an overlaid figure of the fitted half-Gaussian function of the mean rate of the “synchronous” response (Figure 5B) and the mean rate of the “self” response (Figure 8B) against the SD of asynchrony without the reference data points. The multiple comparisons revealed that none of the parameters were significantly different between SJ and AJ under both conditions in both sessions (all *p*s > 0.05).

## Discussion

The present study attempted to clarify whether a modulation of the SoA along with TR is due to a change in the perceptual sensitivity and/or a change in the decision criterion. To determine which component plays a dominant role in the modulation of the SoA with TR, the present study introduced an established experimental paradigm of a sensorimotor coordination task with the SDT (Repp and Knoblich, 2007; Knoblich and Repp, 2009) in combination with a standard adaptation test paradigm to explore the sensorimotor TR (e.g., Heron et al., 2009; Sugano et al., 2010, 2012, 2014).

The participants performed a reproduction task of the sequence of tones with constant ISI by pressing a computer mouse, which triggered a tone. The ISI and the number of tones were varied randomly across trials. In the reproduction task, the computer took over the control of the tones from the participants in 50% of the trials. The participants made a 2AFC judgment about whether the tones were controlled by themselves or by the computer (AJ) and about whether or not the tones were synchronous with their mouse presses (SJ). Before doing these tasks, the participants were exposed to delayed auditory feedback (i.e., 180 ms) during another reproduction task in which they always controlled the tones. The participants also experienced the subjectively synchronous feedback condition (i.e., 80 ms) as a baseline. The delayed feedback and baseline conditions were blocked, and their execution order was counterbalanced across participants.

### Summary of the Results

The present study revealed three main findings. First, as was predicted, exposure to delayed auditory feedback modulated the TWS and caused the TR. The distribution of the mean rate of “synchronous” response against the mean asynchrony under the computer-controlled trials, which can be regarded as the TWS, shifted its center in the direction of the delay. In addition, the TWS widened its width under the delayed feedback condition compared with the baseline condition (Figure 4B). On the other hand, the mean rate of “synchronous” response under the self-controlled trials significantly decreased under the delayed feedback condition compared with the baseline condition (Figure 6). These results clearly indicate that TR occurred: exposure to delayed auditory feedback modulates both the center and the width of the TWS between voluntary action and sensory feedback. This result is consistent with previous research, which has shown the shift of the PSS between action and feedback in the direction of the exposed delay (Stetson et al., 2006; Heron et al., 2009; Sugano et al., 2010, 2012, 2014) as well as decrease in sensitivity for subjective simultaneity between action and feedback after exposure to delay (Winter et al., 2008; Keetels and Vroomen, 2012; Timm et al., 2014; Sugano et al., 2016). Some researchers have argued that widening the TWS is a first step to compensate for the delay (Navarra et al., 2005; Winter et al., 2008). The present results support this claim.

Second, the exposure to delayed auditory feedback also modulated the TWA. The distribution of the mean rate of “self” response against the mean asynchrony under the computer-controlled trials, which can be regarded as the TWA, shifted its center in the direction of the delay (Figure 7B). However, the shift of the midpoint of the TWA was smaller than that of the TWS and was not statistically significant. On the other hand, the mean rate of “self” response under the self-controlled trials significantly decreased after exposure to delayed auditory feedback (Figure 9). These results clearly indicate that the SoA over the auditory feedback decreased after exposure to the delay, which is consistent with previous findings (Timm et al., 2014; Imaizumi and Asai, 2015; Imaizumi and Tanno, 2019). However, the modulation of the TWA was slightly different from that of the TWS: neither the midpoint nor the width of the TWA was significantly different between the delayed condition and the baseline condition in the second session (Table 3), suggesting that the TWA was modulated to a lesser degree by the delay exposure as compared to the TWS. I will discuss a possible explanation of this difference between the TWS and the TWA in the following section.

Third, and critically, the SDT analysis of the AJ data revealed that the degraded SoA after exposure to the delay was caused by the change in response bias without a loss of perceptual sensitivity of agency (Figure 10A). The intact sensitivity of the agency corresponds well with the unchanged width of the TWA between the baseline and the delayed conditions in the second session (47.2 vs. 44.7 ms; Figure 7B and Table 3). Similarly, the change in the response bias corresponds to the shift in midpoint of the TWA between the baseline and the delayed conditions (−118.6 vs. −135.6 ms; Figure 7B and Table 3), although it approached significance but was not significant (*p* = 0.080). Thus, the results were coherent and showed no contradiction.

What psychological mechanism underlies the degraded SoA along with the TR? Within the framework of the hypothetical model of the sensorimotor coordination and agency perception, the modulation in the comparator mechanism between the predicted state and the actual state might be the one (Frith et al., 2000; Synofzik et al., 2008). The discrepancy between the predicted state and the actual state would trigger a continuous recalibration process of the action predictions and motor behavior (Synofzik et al., 2006) to balance the exactness of the prediction and the tolerance zone of when and where the sensory feedback is perceived (Synofzik et al., 2008). Comparing the modulation of the TWA against that of the TWS, I will discuss in detail how the comparator relates to the SoA modulation later.

There are two additional findings in the present study. First, the participants used the fluctuation of asynchrony in a series of action–feedback pairs as a sensorimotor cue for SJ and AJ. However, the exposure to delayed auditory feedback did not have a significant impact on their manner of using the fluctuation cue for SJ (Figure 5B and Table 2) and AJ (Figure 8B and Table 4). In addition, a comparison between the fitted half-Gaussian functions of SJ against the SD of asynchrony and that of AJ revealed that they were similar (Figure 11C), suggesting that the participants used the fluctuation cue in a similar way to judge simultaneity and agency.

Second, the correlational analysis between SJ and AJ revealed that they were similar yet different in several aspects. A majority of the participants judged SJ and AJ differently (Figure 11A), which means that the participants indeed distinguished agency from synchrony, even when they performed SJ and AJ within the same experimental trial. Moreover, the comparison of the estimated shape of the TWS and that of the TWA revealed that the width of the TWA (the “sigma”) was significantly narrower than that of the TWS in both sessions under the delayed condition but not under the baseline condition (Figure 11B), suggesting that they were differently modulated by the exposure to delay.

### Robustness of the Temporal Window of Agency Against the Temporal Recalibration

As shown in Figure 11B, the TWS and the TWA seemed to be slightly different in terms of how they were modulated after exposure to delayed auditory feedback. The width of the TWS became wider after exposure to delay (19.1-ms difference between the baseline and the delayed conditions in the second session; Table 1), while the width of the TWA did not (−2.5-ms difference between the baseline and the delayed conditions in the second session; Table 3). Moreover, the midpoint of the TWS significantly shifted after exposure to delay (−31.8-ms difference between the baseline and the delayed conditions in the second session; Table 1), while the midpoint of the TWA tended to but did not shift significantly (−17.0-ms difference between the baseline and the delayed conditions in the second session; Table 3). These results suggest that the TWA was more robust against the disturbance by the exposure to delay than the TWS.

This seems to be coherent with the earlier findings showing that AJ is less sensitive to the feedback delay than SJ. The TWA is wider than the TWS for the action-lead side (Rohde et al., 2014b). The intentional binding (Haggard et al., 2002b), which is thought to be an implicit measure of the SoA, can occur up to 650 ms (Haggard et al., 2002a) or even up to 4,000 ms (Humphreys and Buehner, 2009). The explicit judgment of agency is less sensitive to the delay in the outcome even up to 600 ms after the action (Karsh et al., 2016).

However, earlier empirical research, which directly explored the modulation of the SoA by TR, did not reach consensus as to whether the TWA is more robust against the disturbance by the exposure to delay than the TWS (Timm et al., 2014; Imaizumi and Asai, 2015). Imaizumi and Asai (2015) reported similar findings demonstrating that the decrement in the agency rating after exposure to delay tended to be smaller than that of the synchrony rating, although it was not statistically tested. On the other hand, Timm et al. (2014) found that both the psychometric function of agency and that of simultaneity modulated after exposure to delay but did not find a significant difference about the extent of modulation between them. Thus, it should be further tested in future research.

#### Two-Stage Model and Bayesian Framework

The lesser modulation of the TWA than the TWS after exposure to delayed auditory feedback can be explained in terms of the two-step account of SoA by Synofzik et al. (2008) and the Bayesian cue integration framework of SoA proposed by Moore and Fletcher (2012).

The two-step account of SoA assumes two processing stages to generate the SoA: (1) perceptual or sensorimotor integration stage and (2) cognitive or conceptual evaluation stage (Synofzik et al., 2008; Rohde et al., 2014b). The sensorimotor integration stage compares the actual sensory feedback after voluntary action to the predicted one and generates a preconceptual, low-level feeling of agency (FoA). The conceptual evaluation stage then assigns a conceptual judgment of agency (JoA) by integrating the FoA with intentions, thoughts, and various contextual cues. Thus, the SoA is a product of both bottom–up processes (FoA) and top–down processes (JoA) (Synofzik et al., 2008).

Both FoA and JoA are affected by multiple internal and external cues such as sensory feedback, proprioception, motoric cues, intentions, thoughts, and contextual and social cues (Synofzik et al., 2008; Moore et al., 2009; Moore and Fletcher, 2012; Farrer et al., 2013). The Bayesian cue integration framework explains how these cues get integrated and generate the SoA (Moore and Fletcher, 2012). The manner of integration is influenced by the reliability of each cue and is governed by Bayes’ rule.

Using the two-step account of the SoA (Synofzik et al., 2008) and the Bayesian cue integration framework (Moore and Fletcher, 2012), the different modulation by TR between the TWS and the TWA can be explained in terms of different cue integration weights between them. There are at least two possible explanations: (A) the different weights between the sensorimotor cues (e.g., asynchrony between action and feedback) and the contextual cues (e.g., cause–effect relationship) and (B) the different weights between the internal cues (i.e., motoric signals) and the external cues (i.e., sensory inputs). These two explanations are not mutually exclusive.

Explanation (A) assumes that the impact of the “contextual cues” (i.e., cause–effect relationship) on AJ is larger than that on SJ, and that the contextual cues may be modulated to a lesser degree by TR compared to the sensorimotor cues (i.e., perceived simultaneity). The former assumption is derived by the fact that AJ in the present study was the explicit JoA, which is thought to contain an attribution process in decisional processing level and, therefore, might be influenced more by the contextual cues than the sensorimotor cues (Synofzik et al., 2008). In addition, although SJ is influenced by both sensorimotor cues and contextual cues (Haggard et al., 2002b; Buehner and Humphreys, 2009; Bechlivanidis and Lagnado, 2013), it is reasonable to assume that it relies more on the sensorimotor cues (i.e., perceived simultaneity) than the contextual cues (i.e., cause–effect relationship). The latter assumption is supported by the empirical finding that the cause–effect relationship can be maintained even if the sensory feedback is delayed up to 650 ms (Haggard et al., 2002a) or even up to 4,000 ms (Humphreys and Buehner, 2009). With these assumptions, the relatively smaller influence of the TR on the TWA than on the TWS can be explained.

On the other hand, explanation (B) assumes that the impact of the “internal cues” (i.e., motoric signals) on AJ is larger than that on SJ and that the internal cues may be modulated by the TR to a lesser degree than the external cues (i.e., sensory inputs). The former assumption is derived from the earlier suggestion that the internal cues are the most reliable and are the strongest contributor to the SoA (Moore and Fletcher, 2012; Wen, 2019). However, it is worth noting that there is still a controversy regarding whether or not sensory feedback is necessary for the SoA (e.g., Synofzik et al., 2008; Carruthers, 2012). The latter assumption is supported by the empirical finding that the timing of the sensory feedback is shifted more than that of the voluntary action in the intentional binding (Haggard et al., 2002b). With these assumptions, the relatively smaller influence of the TR on the TWA than on the TWS can be explained as well.

#### Why Was the Temporal Window of Agency Narrower Than the Temporal Window of Simultaneity?

As described earlier, the present study showed that the TWA was significantly “narrower” than the TWS under the delayed condition (Figure 11B). However, one might suspect that the TWA should be “broader” than the TWS, as the contextual cues such as the cause–effect relationship might facilitate the SoA (e.g., Rohde and Ernst, 2012). In fact, Rohde et al. (2014b) found that the width of the TWA was more than that of the TWS, though it was specific to the movement-lead-flash side. How can we reconcile this discrepancy?

It is likely that the task difference between the current study and Rohde et al. (2014b) might play a role. In the present study, the initial two feedback sounds in the test phase were always self-controlled and were presented with a fixed 80-ms delay regardless of the conditions. Therefore, the existence of the delay in the subsequent sounds was a strong cue for an absence of self-control. As a result, the participants in the current study would be more sensitive to the delay in the AJ task than those in Rohde et al. (2014b).

In addition, the difference in the experimental design might also play a role. In Rohde et al. (2014b), the SJ and the AJ were executed in separate experimental trials, and thus either of the tasks might be equally focused upon by the participants. However, in the present study, SJ and AJ were mixed within a single trial and the AJ always precedes the SJ. Furthermore, the confidence rating was required only for AJ but not for SJ. With these differences, the participants might focus on AJ more than SJ, making them more sensitive to AJ than to SJ in the current study and possibly leading to the narrower TWA than the TWS.

Furthermore, it has been pointed out that the SJ task is not the best way to measure the width of the TWS, as it is vulnerable to the participant’s decision criterion for synchrony and to cognitive biases (e.g., unity assumption) (Vroomen and Keetels, 2010). With this in mind, the width of the TWS in the current study may possibly be overestimated. Therefore, the question regarding the relative widths of the TWA and the TWS is still open to be answered.

### Conclusion

In sum, the present study found that both TWS and TWA were modulated after exposure to delayed auditory feedback. However, the TWA was modulated by the TR to a lesser degree than the TWS. Critically, the degraded SoA by the TR is due to the change in decision criterion but not in the perceptual sensitivity for agency, suggesting that it would be a product of the late stage of processing.

## Data Availability Statement

The datasets presented in this study can be found in online repositories. The names of the repository/repositories and accession number(s) can be found below: https://doi.org/10.6084/m9.figshare.12607697.

## Ethics Statement

The studies involving human participants were reviewed and approved by the Local Ethics Committee of Kyushu Sangyo University. The patients/participants provided their written informed consent to participate in this study.

## Author Contributions

YS designed the study, performed the experiment, analyzed the data, and wrote the manuscript.

## Conflict of Interest

The author declares that the research was conducted in the absence of any commercial or financial relationships that could be construed as a potential conflict of interest.
